# The Onset of a Peripheral Ameloblastoma

**DOI:** 10.1155/2012/729467

**Published:** 2012-05-17

**Authors:** Kellen Cristine Tjioe, José Humberto Damante, Denise Tostes Oliveira

**Affiliations:** ^1^Department of Stomatology, Bauru School of Dentistry, University of São Paulo, Bauru, SP, Brazil; ^2^Area of Pathology, Department of Stomatology, Bauru School of Dentistry, University of São Paulo, Bauru, SP, Brazil; ^3^Alameda Octávio Pinheiro Brisolla, 9-75, 17012-901 Bauru, SP, Brazil

## Abstract

Incipient odontogenic tumors often display intermediate features between two or more lesions leading to diagnosis dilemma. We report the onset of a peripheral ameloblastoma fortuitously found subjacent to a nondysplastic leukoplakia in the region of missing 38 teeth of a 52-year-old man. The aim of this paper is the discussion of the microscopical features observed in the case reported which allowed the establishment of the final diagnosis of an early peripheral ameloblastoma.

## 1. Introduction

The odontogenesis is a set of complex interactions between the oral epithelium and the ectomesenchyme which culminates with the formation of the tooth. After this process, residual odontogenic epithelial cells remain dormant in the tissue of the jaws indefinitely and may proliferate later, generating cysts and tumors [1]. This process of transformation rarely is documented once usually the neoplasias are excised due to signals and symptoms of the patients, that is, when the lesion is well established. Furthermore, in the initial phase of development, the limit between a true neoplastic and a hamartomatous lesion has not a definitive border line and may represent a challenge of diagnosis.

Therefore, the aim of this work is to present the onset of a peripheral ameloblastoma fortuitously found subjacent to a nondysplastic leukoplakia, discussing the microscopical findings which led us to its final diagnosis.

## 2. Case Presentation

**Figure 1:**
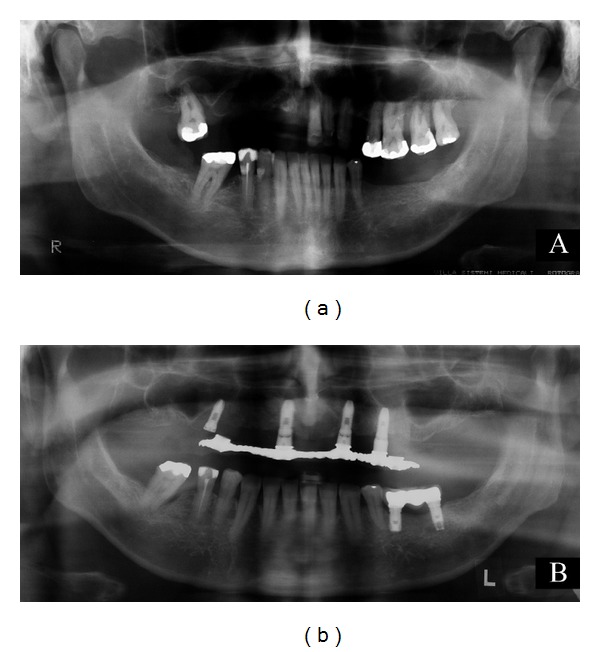
Panoramic radiographs of the patient. (a) Taken at the first visit. No bone alterations can be seen in the area of the lesion; (b) Nine-year follow-up: no change worthy of note.

A 52-year-old man sought for treatment of a white plaque in the gingiva. The lesion was asymptomatic and had been noticed by the patient two years before the consultation. He reported daily tobacco and alcohol consumption. Oral examination revealed an 8 mm diameter and ill-defined white plaque on the edentulous alveolar ridge, region corresponding to the 38 tooth. Radiographs showed normal bone appearance (Figure 1(a)). The clinical diagnosis was leukoplakia. 

**Figure 2:**
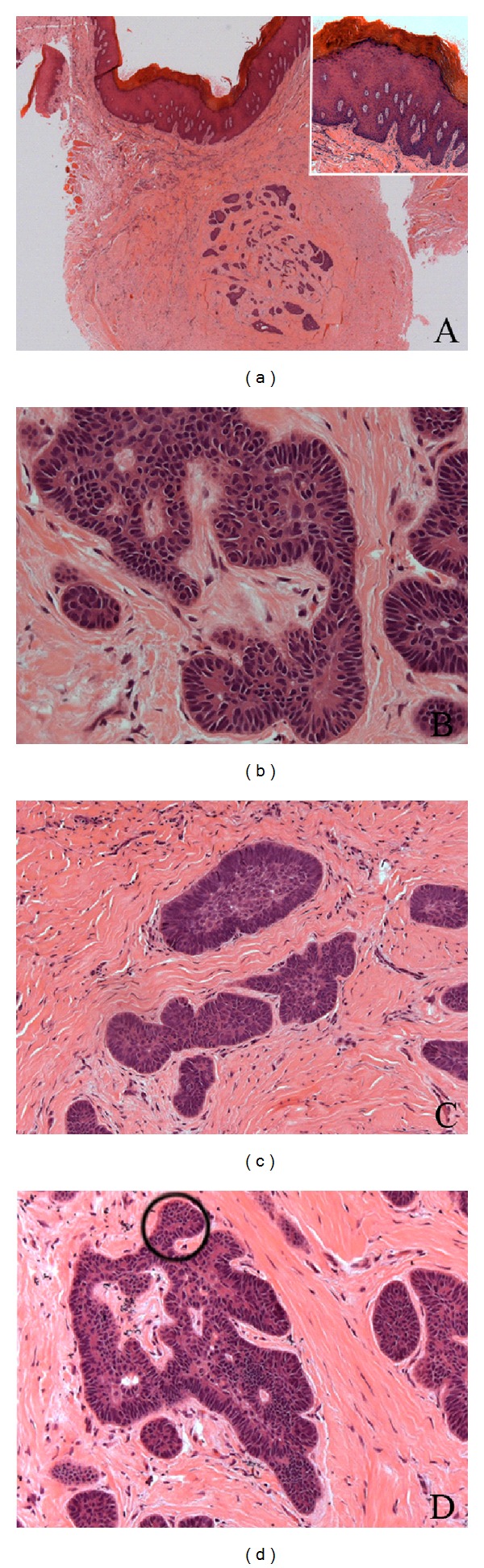
(a) Nondysplastic hyperkeratotic oral epithelium (magnified in the box, H&E, 10x) and the submucous odontogenic epithelial cell islands (H&E, 2,5x); (b) Less mature odontogenic island with vacuolated oval to polyhedric cells (H&E, 40x); (c) ameloblastomatous nest composed by peripheral palisading columnar and central polyhedric cells (H&E, 20x); (d) budding projection in an ameloblastomatous island (circle) (H&E, 20x).

**Figure 3:**
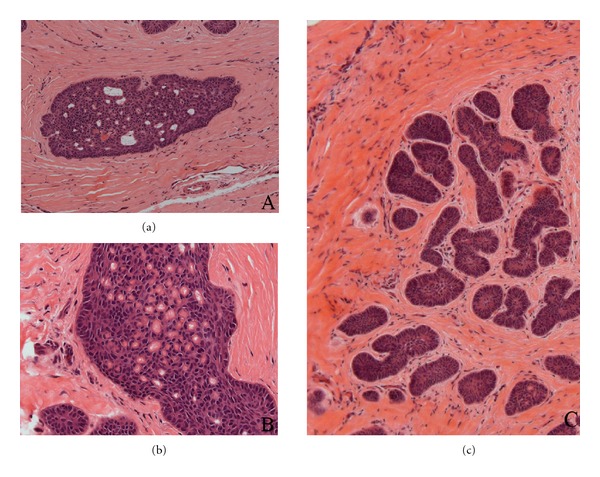
(a) Cystic formations in an ameloblastomatous nest (H&E, 20x); (b) squamous metaplasia seen in an ameloblastomatous nest (H&E, 20x). (c)Stroma surrounding the odontogenic epithelial islands with a more fibrous arrangement than that outer connective tissue (H&E, 10x).

Under local anaesthesia, the white plaque was excised. Microscopically, the oral squamous epithelium was hyperplastic and hyperkeratotic but without dysplastic alterations (Figure 2(a)). A discrete chronic inflammatory infiltrate in the subjacent connective tissue was observed. In the deep portion of oral submucosa there were numerous islands of odontogenic epithelial cells scattered in a fibrous stroma (Figure 2(a)). Few nests were predominantly composed by polyhedric cells (Figure 2(b)). Other epithelial islands consisted of peripheral rows of palisaded hyperchromatic columnar cells and central polyhedric cells. The outer cells presented more basophilic staining than that inner ones (Figure 2(c)). In few larger islands, it was possible to see evident budding projections (Figure 2(d)). Semiserial section of the lesion exhibited epithelial islands with squamous metaplasia and incipient cystic formation (Figures 3(a) and 3(b)). Another interesting microscopical feature was the presence of connective tissue surrounding the islands of odontogenic epithelial cells with looser arrangement than adjacent collagenous tissue not involved with the lesion (Figure 3(c)). The aggregate of odontogenic epithelial cells was confined to the connective tissue, without bone infiltration. The diagnosis established was early peripheral ameloblastoma subjacent to a nondysplastic leukoplakia. The patient was submitted to a long follow-up. In the nine-year follow-up, he was clinically and radiographically normal (Figure 1(b)). 

## 3. Discussion

The odontogenesis is an intricate process by which the formation of the tooth occurs. After its conclusion, residual epithelial cells remain scattered in the soft tissue adjacent to the tooth. In some cases, it stays dormant indefinitely but in others, it may proliferate and raise odontogenic cysts or tumors [1]. Nevertheless, diagnostic criteria for identifing incipient lesions have not been established yet, despite of the efforts for this purpose [2]. In this context, one of the greatest challenges of the diagnosis is given by lesions with intermediary characteristics between hamartomatous and neoplastic diseases. 

The presented case illustrates an uncommon occurrence: the onset of an odontogenic tumor. It is impossible to anticipate the development of this accidentally found tumor supposing no concomitant appearance of this with the leukoplakia, which motivated the patient to seek for treatment. However, details concerning the microscopical characteristics and radiographic images observed in the case allow us to reach some interesting conclusions. 

The peripheral ameloblastoma is the extraosseous counterpart of the conventional ameloblastoma. Therefore, they share the same microscopical features defined by the World Health Organization [3], as follows: (1) islands with peripheral columnar cells surrounding central ones resembling the stellate reticulum; (2) the peripheral cells are hyperchromatic, lined up in a palisaded fashion, their nuclei are displaced away from the basement membrane and the cytoplasm are vacuolated; (3) the central cells are loosely arranged and often become cystic [3]. However, these classic characteristics may not be observed in few cases, including incipient lesions [2]. Looking for the delineation of histopathologic features of early ameloblastoma, a classic work performed by Vickers and Gorlin [4] gave birth to the so-called *Vickers and Gorlin criteria*, which is still largely utilized. *Vickers and Gorlin criteria *state that nuclear hyperchromatism, nuclear palisading with reverse polarization, and cytoplasmic vacuolization with intercellular spacing, when observed together, constitute histopathologic evidence of neoplasia [4]. Our case does not fulfill all the features required by *Vickers and Gorlin criteria *to diagnosis of ameloblastoma, as reverse polarization could not been seen. However, it does not necessarily mean that the peripheral ameloblastoma is discarded from the list. An elegant discerning review of some controversial aspects of ameloblastoma performed by Gardner [2] drew attention to the fact that not all ameloblastomas exhibit classic *Vickers and Gorlin criteria*. Therefore, the cytoplasmic vacuolation and reverse polarity may not be present but the basal cells should be palisaded, columnar, and hyperchromatic [2], as was exactly observed in the presented case. It confirms the diagnosis of peripheral ameloblastoma. Moreover, we agree with Gardner [2] when he says that although the *Vickers and Gorlin criteria* were originally described to help diagnose early ameloblastomas in cysts, they are useful when examining other lesions that are suspected of being ameloblastomas [2]. In other words, the *Vickers and Gorlin criteria* are indeed helpful for differential diagnosis, but not enough to establish the diagnosis of ameloblastomas in very incipient lesions.

Furthermore, the bud projections mimicking the normal embryologic development of the tooth bud at the stage of enamel matrix production [5] and common in ameloblastomas, have been found in our case. Still important is the difference between the connective tissue surrounding the lesion and that not involved with this. It is believed that the ameloblastic epithelium, in an attempt to complete its embryologic function and produce enamel matrix, signals the connective tissue to induce dentin formation; however, the cells in the connective tissue are unable to respond appropriately, resulting in hyalinized zones [5] (Figure 3(c)). Finally, the hamartomas are lesions which develop during the growth period and the patient was on the sixth decade of life, another fact pointing to the neoplastic nature of the lesion. Thus, based in the microscopic aspects, the final diagnosis established was early peripheral ameloblastoma. In the presented case, the precocious finding of the fortuitously removed ameloblastoma enabled us to establish the long-term follow-up of the patient in order to prevent underdiagnosis of any possible recurrence or complication. In addition, this report emphasizes the importance of the histopathologic analysis for early diagnosis of neoplastic lesions in uneven oral mucosa.
